# Feel Good? The Dialectical Integration of International Immigrants in Rural Communities: The Case of the Canadian Prairie Provinces

**DOI:** 10.3389/fsoc.2020.578076

**Published:** 2021-01-18

**Authors:** Jennifer Dauphinais, Sherine Salmon, Mikaël Akimowicz

**Affiliations:** ^1^Rural Development Institute, Brandon University, Brandon, MB, Canada; ^2^Laboratoire d'Etude et de Recherche sur l'Economie, les Politiques et les Systèmes sociaux, Université Toulouse III – Paul Sabatier, Toulouse, France

**Keywords:** international immigration, immigrant integration, socialization, rural identity, well-being, Canadian Prairie provinces

## Abstract

The increasing influx of international immigrants settling in rural communities, where their landing is expected to revitalize communities, has triggered concerns about international immigrants' adaptation and well-being. In this article, we specifically focus on international immigrants' economic integration as a part of their socialization in communities. This article integrates the results of two independent studies, respectively, focusing on rural employers' motivations to hire immigrants and immigrants' integration in rural communities, both taking place in the Canadian Prairie provinces. Based on a survey of 112 employers and 36 in-depth interviews with international immigrants and organizations promoting their integration, we explore the impact of mediating organizations on the well-being of international immigrants. The results highlight that mediating organizations facilitate the sharing of meanings between rural communities' stakeholders, which is key to success for both employers and employees in formalized organizations such as businesses. The results suggest that international immigrants' well-being is facilitated by mediating organizations that foster a dialectical transformation of rural communities where both hosts and immigrants understand each other.

## Introduction

Canada's appeal has fostered strong immigration flux, be it to escape perilous situations, to discover new beginnings, or to experience financial prosperity (Gignac, 2013; Randstad, n.d.). Immigration has become a primary driver of population growth throughout the country. In 2018, international in-migration accounted for 82% of the Canadian population increase (Statistics Canada, 2019) and the immigrant population represented 22% of the Canadian population, the highest since the 1921 census (Statistics Canada, 2017a). In 2018, the number of international immigrant entrances reached 321 035, placing Canada's population growth in first place among its G7 counterparts (Statistics Canada, 2019). In a context of an aging population (Coutinho, 2018; Statistics Canada, 2018), Canada stands to benefit from the ongoing influx of younger immigrants. This is especially true for rural communities, where population aging, youth retention, and labor shortages are major challenges (Aure et al., 2018; Immigration, Refugees and Citizenship Canada, 2019a).

Regionalization policies (e.g., the Provincial Nominee Program launched in 1996 or the Local Immigration Partnerships launched in 2008) have seen the redistribution of immigrant populations away from the larger gateway cities and toward smaller cities and rural areas of the country (Brown, 2017). By doing so, population growth is now more spread out, helping to reduce population decline in smaller communities, while helping to alleviate the growth pressures placed on larger cities' centers (Carter et al., 2008). Although broader regional distribution can help to revitalize declining communities, it can also bring with it some challenges. Despite the number of immigrants settling outside of larger metropolitan areas, the organizations serving these communities are often underrepresented. This has implications for immigrants who may not be able to access government-mandated services that are unevenly distributed across rural municipalities (De Lima and Wright, 2009; Mukhtar et al., 2016; Arora-Jonsson, 2017).

Leveraging immigration for the revitalization of rural communities requires welcoming international immigrants in a manner that enables them to find their place socially, culturally, and economically (Carter et al., 2008; Wulff et al., 2008; Brown, 2017). Thus, there is a need to better understand the complex adaptation dynamics of international immigrants in rural Canada.

For Shields et al. (2016), the process of settlement targets adjustment (acclimatizing to the language, culture, environment), adaptation (mastering situations without great assistance), and integration (the ability to contribute to the host society free of social, economic, cultural, and political barriers). Settlement organizations are expected to assist immigrants to the point of integration and citizenship through direct (i.e., needs assessment and referrals, information and orientation, language training, employment-related, community connections) and indirect services (i.e., partnerships and capacity building) (Immigration, Refugees and Citizenship Canada, 2019c). A dense network of settlement organizations is an integral part of Canada's strategy, which is generally positively acknowledged globally (Sidney, 2014). However, studies such as Kaushik and Drolet (2018: p.2), who pointed out “disappointing results in the economic and social outcomes of the integration of skilled immigrants,” tend to show that Canada's current strategy can be improved. Box 1 provides a historical overview of the Canadian immigration policy. In particular, social and health policy-makers should consider strategies to increase access to culturally and linguistically appropriate services (Mental Health Commission of Canada (MHCC), 2019). While Canada has focused on the successful tenets of integration, immigrants' well-being is often neglected or approximated by economic measurements (Frank et al., 2014; Canadian Index of Wellbeing, 2016; De Lima et al., 2017).

Box 1 **Historical overview of the Canadian Immigration Policy.**

Canada's multifaceted immigration policy integrates social (i.e., reuniting families), humanitarian (i.e., providing a safe haven), and economic (i.e., recruiting workers) aspects of life (Parliament of Canada, 2015). However, Sharma (2000: p.6) notes that it is framed by “regulations governing the movement of people into Canada, as well as the legislation on citizenship, [that] have historically shaped both the territorial boundaries of the Canadian nation and people's consciousness about'being Canadian.” In particular, “state laws on citizenship and immigration have helped to organize a hierarchical ordering of insiders and outsiders living and working within Canadian society” (Baines and Sharma, 2002: p.85).

Since the formation of the Dominion of Canada, restrictions on entry applied based on country of origin and ethnicity, criteria later reasserted in several Immigration Acts such as in 1910 and 1952 (Hawkins, 1991; Walsh, 2008). The official dismantling of discriminating Eurocentric policies began in 1962, replaced by an economic emphasis on human capital leading to the development of a point system in 1967 (Walsh, 2008). In 1973, a Non-Immigrant Employment Authorization Program introduced a migrant worker class (Sharma, 2001). Nowadays, individuals can be distinguished into migrant workers,^1^ permanent residents, and citizens.

A network of settlement organizations—monoethnic, multicultural, faith-based, specialized, or a function of a broader mandate in an organization—has been developed (Canada Council for Refugees, 2000). Prior to World War II, settlement organizations existed informally through faith-based or ethnic organizations. After World War II, the influx of international immigrants initiated both (1) the provision of more developed services by private organizations that catered not only to physical needs but also to mental health needs for war victims, and (2) the development of the Citizenship Branch in the Department of Citizenship and Immigration by the Canadian government.^2^ Subsequently, provincial and municipal governments formed departments to support or fund settlement programs.

Well-being, defined as “the highest possible quality of life in its full breadth of expression,” focuses on “but [is] not necessarily exclusive to, good living standards, robust health, a sustainable environment, vital communities, an educated populace, balanced time use, high levels of democratic participation, and access to and participation in leisure and culture” (Diener et al., 2010; Wells, 2010; Canadian Index of Wellbeing, 2016: p.11). According to the Mental Health Commission of Canada (2019), immigrants arriving in Canada are usually healthier (mentally and physically) than their Canadian counterparts; however, there is a decline in health after 5 years. Furthermore, immigrants are less likely to seek mental health support because of barriers such as language, stigma, and fear stemming from discomfort or unfamiliarity (Robert and Gilkinson, 2012; Mental Health Commission of Canada, 2019). Employment is a salient feature in understanding how immigrants feel about life in Canada as they tend to adjust better in the society when they are more satisfied with their employment (Richmond, 1974; Starr and Roberts, 1982; Zhou, 1997): “alienated immigrants whose failure to obtain steady employment at a level commensurate with their qualifications combined to social isolation and lack of acculturation generate deep-seated dissatisfaction” (Richmond, 1974: p.47). As immigrants' well-being contributes to the social cohesion of the society at large, it is critical to shed light on the articulation of immigrants' well-being and their economic integration (Aycan and Berry, 1996; Robert and Gilkinson, 2012; Berry and Hou, 2016; Toronto Region Immigrant Employment Council, 2018).

With a focus on the Prairie provinces of Manitoba, Saskatchewan, and Alberta, where the number of immigrants doubled between 2001 and 2016 (Statistics Canada, 2017a) and represented the landing point of 27% of Canada's immigrants in 2017 (Statistics Canada, 2017b), the goal of this article is to examine the role played by mediating organizations such as settlement organizations, i.e., third-party intermediaries that contribute to facilitating international immigrants' integration by helping them to make sense of their new environment on the well-being of international immigrants in rural areas. International immigrants' integration process is conceptualized as a secondary socialization, as defined by Berger and Luckmann (1966), a theoretical choice that emphasizes the cognitive dimension of the integration process, which goes beyond previous authors' emphasis on racism and discrimination. In particular, it enables a meso-level analysis that focuses on interactions between social actors. It is in line with Baines and Sharma's (2002: p.98) recommendation to focus on “grassroots sense of building community through the recognition of and support for the struggles and goals of differently empowered, local, and global communities of interest.” The focus is on the Canadian Prairie provinces where the energy and the agri-food sectors attracted immigrants, who represent ~8% of the regional population, the highest share in Canada. While ethnicity had shaped vibrant communities during the XIX century, nowadays these communities may struggle with the integration of the recent arrivals of international immigrants due to a hierarchical ordering of insiders and outsiders, as described by Baines and Sharma (2002). This study also focuses on small and medium enterprises (SMEs), which comprise 98% of Canadian businesses and employ 91% of the labor force in the Canadian prairies (Statistics Canada, 2016a). Despite notable labor shortages−39% of SMEs reported difficulty in getting new hires—SME entrepreneurs were less likely to hire immigrants (Business Development Bank of Canada, 2018). Finally, a combination of quantitative and qualitative data extracted from a survey of SME employers and interviews with both immigrants and settlement organizations is used to triangulate immigrants' experiences with employers' and settlement organizations' practices. The results show that the most successful integration takes place when there is a mutual secondary socialization of both hosts (i.e., welcoming communities) and newcomers (i.e., international immigrants), a process that we labeled dialectical integration. Interestingly, in the current neoliberal regime that sees cuts in the means of traditional settlement organizations, SMEs play a critical role in this process of dialectical integration.

The following section presents the conceptual framework designed for this analysis, which mixes Berger and Luckmann's (1966) secondary socialization concept with Porter's (2018) health perspective. We then expose the methods and data used for the analysis, which blend two independent researches with complementary perspectives. The results are presented in the section that follows and highlight the critical role of mediating organizations in immigrants' socialization process. Finally, after discussing the results, we conclude this article.

## Literature Review

This literature review builds on Berger and Luckmann's (1966) secondary socialization concept—i.e., the idea that “there is a temporal sequence, in the course of which he [the individual] is inducted into participation in the social dialectic” (Berger and Luckmann, 1966: p.149). In the context of international immigrants' integration in rural areas, we first show that the emergence of a sense of place is contingent on the stabilization of interdependent shared meanings before incorporating Porter's (2018) health perspective to tackle international immigrants' well-being. The neoliberalization of immigration policies is finally highlighted as a potential threat to the integration of immigrants, hence to their well-being.

### Socialization and Sense of Place

Prevailing cultural ideologies of rural communities are usually heavily laden with notions of rootedness, localism, stability, and attachment to place (Milbourne and Kitchen, 2014; Lysgård, 2019). Whether materially true or not, they shape the symbolic representation of rural communities. At the same time, they also create a tension between locals and newcomers as the “authentic” countryside is perceived as disappearing, and the culture and linguistics are changing with it (Milbourne and Kitchen, 2014; McAreavey and Argent, 2018). In line with the official state discourse that fuels an insider/outsider comprehension of immigration issues, rural inhabitants' struggles for the means of production and reproduction may result in the perception of being threatened by needy newcomers (Sharma, 2000; Brown, 2017). While out-migration is generally the accepted response to rural problems such as poverty and homelessness, when poor individuals seek to relocate to rural areas, their presence is often contested by the local community (Milbourne and Kitchen, 2014). Further, the redistribution of public and social services to the more populated urban centers and the deterioration of public transportation leave many people stranded within local spaces and force others to employ complex strategies to access facilities and services that have relocated to other places (Milbourne and Kitchen, 2014).

Despite settlement policies that support immigrants' retention to a certain place, there are still a number of barriers that hinder well-being (Wulff et al., 2008). The integration of immigrants into a rural or small town is contingent on their acceptance into the community. One's sense of community may be threatened by the presence of outsiders that represent a connection to the change happening both within and outside of the broader community (Amsden et al., 2010; Radford, 2016). As immigrants' status changes from one of outsider to one of insider, the idea of community begins to change and is manifested in a “sense of us” that is collectively created through interaction, shared identity, and shared experiences (Amsden et al., 2010; Radford, 2017), paving the way for the development of a sense of place, “a complex creation of social and individual interactions and meanings that inform how people perceive the world around them. Community, as a unit of analysis, is a construction people use to organize these social interactions in a meaningful way” (Amsden et al., 2010: p.33). Contrary to the segmented assimilationist view that the white urban population is perceived as a cultural reference, we follow García and Schmalzbauer (2017) that assimilation is framed by the demographic populations as well as the physical geography of the welcoming communities.

*Hypothesis 1: The denser the social network of immigrants in the host society, the easier their navigation of the social worlds*.

### Sense of Place and Well-Being

In this article, the conceptualization of well-being focuses on the mental, emotional, and social aspects of individuals' life as they become integrated within a community. During a socialization process, well-being is more than just a by-product of health; it becomes articulated through individuals' capacities to function well within their respective communities, along with their ability to engage in the process of self-actualization and their own personal development (Maslow, 1943; Porter, 2018). In this case, Porter's (2018) concept of embodiment can be applied to struggles experienced by immigrants as they attempt to integrate into their new surroundings. Through this concept, well-being articulates social functions as well as personal feelings, becoming an individual and social phenomenon that focuses on bodies and bodily interactions. Individuals incorporate and express their social and material worlds; therefore, their desires, activities, and self-interests should not be assumed; nor are they necessarily held in common with others but rather contingent on local bodily practices and relations (Lock, 1993; Csordas, 1994; Mol, 2002; Lock and Farquhar, 2007; Porter, 2018).

A part of self-actualization requires the ability to think across embodied differences. On the one hand, such differences can create exclusionary practices that run counter to prevailing understandings of integration and well-being (Gammeltoft, 2008; Porter, 2018). In this context, the creation and maintenance of well-being are construed as one's ability to make sense of the corporeal responses to the material and social surrounding worlds, to have some understanding of how bodies mutually perceive and affect one another within these contexts (Lundberg, 2000; Porter, 2018). In other words, we define sensemaking as the process of knowledge creation that helps to navigate social and material worlds (Maitlis and Christianson, 2014, Sandberg and Tsoukas, 2020). In these worlds, beings are implicated with one another, informing and challenging their perceptions and experiences in shared spaces (Berger and Luckmann, 1966; Porter, 2018). The resulting worldviews are central to make sense of the world. Therefore, care becomes relational. It becomes the ability to negotiate the different ways that individuals experience and organize their integration into a community along with the ability to compromise between the values and expectations that individuals have (Hosnedlová, 2017; Porter, 2018). Therefore, it requires a certain juxtaposition of the relationships between sites: care between employers and immigrant employees; settlement organizations and regulating governing agencies; the attitudes of the rural communities receiving immigrants, as well as care for the self (Porter, 2018; Friese and Latimer, 2019).

*Hypothesis 2: The more interdependent the social interactions of international immigrants, the more well-being they experience*.

### The Neoliberal Threat

Canadian public policies are ingrained with the neoliberal praxis (Baines and Sharma, 2002; Mukhtar et al., 2016). As a result, the marketization of social service provision, the devolution of government responsibilities in social welfare production to non-governmental actors, and the promotion of free-market values in the non-profit sector have become pervasive in the restructuring of social welfare policies (e.g., cutbacks in services offered, devolution of services to for-profit or nonprofit agencies, and deregulation) (Weaver et al., 2010; Mukhtar et al., 2016). Moreover, the top-down approach utilized for settlement policy (e.g., funding and mandates federally, additional mandates and program development provincially, and programs delivered at municipal or non-profit level) is not always effective. The lack of funding available to settlement agencies increases the pressure to serve more individuals with fewer resources. This naturally leads to program cutbacks, which can be particularly detrimental when it affects programs, such as domestic violence services for women, youth programs, mental health care services, and legal services (Mukhtar et al., 2016). Last but not least, funding usually happens through yearly contracts, which limits the ability to plan for long-term services. Therefore, many of the programs offered become aligned more with the funder's requirements and less about the newcomers' needs (Mukhtar et al., 2016).

Geographical barriers also pose a challenge for the uptake and utilization of settlement services, because of either non-existent or failing public infrastructure, or the location of agencies (Mukhtar et al., 2016). Thus, a broader regional distribution of immigrants carries with it a cost. For instance, the infrastructure and social support networks need to be in place in smaller communities; diversity issues need to be addressed; and, beyond job opportunities, there needs to be a general welcoming feeling, especially as many newcomers will not have the social support networks of a larger ethnic or social group they can join (Carter et al., 2008). Although municipalities have no legislative authority in the area of immigration, many have introduced local strategies and programs to attract newcomers and facilitate their integration (Carter et al., 2008). Apart from municipalities, there are plenty of community, faith-based, and ethnocultural organizations across Canada that work to support and help immigrants with the transition process, such as finding suitable housing to accessing health care, employment, recreational services, and education (Carter et al., 2008).

*Hypothesis 3: In rural areas, a diversity of formal and informal organizations substitute/complement governmental settlement organizations*.

## Methods and Data

This section draws on the results of two research studies, which both took place in the Canadian Prairies and respectively investigated the motivations of rural SME employers to hire international immigrants and international immigrants' integration in rural communities. The two datasets show meaningful overlaps; their combination contributes to the understanding of international immigrants' well-being in rural areas. In this article, we mostly use the qualitative data of the two aforementioned research studies to better understand the role of mediating organizations in the creation of shared meanings among international immigrants, rural SME employers, and settlement organizations. Because only permanent residents can access settlement services, we focus on international immigrants who have been granted permanent residency to analyze the interactions that have been forged with mediating organizations. Since 2016, ~60% of the immigrants who have been granted permanent residency in Canada have entered through the economic class (Statistics Canada, 2017a; Immigration, Refugees and Citizenship Canada, 2019b), which is consistent with the objective to shed light on economic integration. In this section, we present the methods used in each study before detailing the research field, the dataset, and the two samples.

### Methods

The SME employers' motivations study consisted of an online survey made of three sets of questions (Salmon et al., 2019). A first set consisted of seven-point Likert-scale questions investigating the motivations of rural SME employers to hire international immigrants. A second set of four open-ended questions collected information about benefits of hiring immigrants, challenges for hiring immigrants, employers' actions to retain newcomers, and employers' needs for hiring immigrants. A third set gathered characteristics of SMEs and SME owners.

**Figure 1:**
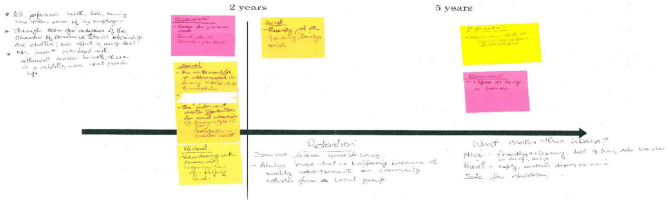
C-IMM2's timeline.

The integration study aimed to compare the integration dynamics of newcomers, defined as both international immigrants and Canadian residents, in rural communities. For the sake of this article, only the data concerning international immigrants are mobilized. It consisted of in-depth semidirected interviews, which lasted, on average, 1 h, with immigrants who had settled in the rural Canadian Prairies as well as with organizations that support immigrants' landing in rural communities. For immigrants, the interview was based on the premise that each integration trajectory is individualized. Immigrants were differentiated based on their seniority in the community (landing took place within the last 2 years, between 2 and 5 years ago, or more than 5 years ago). One representative of each category was interviewed in each community. The research team asked participants to design a timeline highlighting the steps that they perceived as meaningful in their integration process (economic, social, health, political, and educational experiences). These data collection tool was inspired by mental mapping techniques and grounded theory approaches (Akimowicz et al., 2016). Figure 1 below shows an example of the timelines that were collected. Based on the assumption that settlement organizations had a sort of standard procedure to welcome newcomers, the data collection procedure for organizations was initially similar to the one of newcomers. However, the research team quickly realized this was not the case and instead used an analysis grid that enabled the characterization of organizations' actions during the settlement process. This grid was composed of four blocks, which respectively tackled the goals sought by organizations, the related activities implemented by organizations, the feedback received from newcomers, and the opportunities and threats faced by organizations.

### Data

Both studies were conducted in the rural Canadian Prairies. The data for the rural employers' motivations study were collected between August and December 2018, whereas the data for the integration study were collected between July and September 2019. The Canadian Prairies is a vast geographical area delineated by the Rocky Mountains at the west and the Canadian Shield at the East. It is administratively divided into three provinces, namely, Alberta, Saskatchewan, and Manitoba from West to East. The region is economically specialized in the agricultural production of oilseed and grain with some intensive meat production in the three Provinces and extensive ranching in Alberta. The energy industry, while present all over the Canadian Prairies, is particularly developed in Alberta. In 2016, the three provinces of Alberta, Manitoba, and Saskatchewan had the highest shares of population born outside of Canada with an average of ~8% of their population (Statistics Canada, 2016b). Moreover, the largest visible minorities^3^ in the Prairies were the Filipinos, the southern Asians, and the Blacks, who, respectively, represented 37, 16, and 15% of non–metro-visible minorities outside of census metropolitan areas (Statistics Canada, 2016c).

Statistics Canada's definition of rural was used; it excludes large urban population centers exceeding 100,000 inhabitants, which in our case led to the exclusion of Calgary, Edmonton, and Red Deer in Alberta, Regina and Saskatoon in Saskatchewan, and Winnipeg in Manitoba. The low population of the rural Canadian Prairies, 3 million inhabitants, is aging with limited incomes. In this context, immigration is envisioned as a lever to revitalize rural areas with the settlement of new populations. In particular, efforts are made to retain immigrants in rural areas, which is highlighted by Manitoba's Provincial Nominee Program and the launch in 2019 of the Rural and Northern Immigration Pilot. The rural Canadian Prairies therefore provide an interesting case study for unpacking the question of international immigrants' well-being.

The online survey of the SME employers' motivations study was primarily advertised by the service provider organizations (SPOs), which are local settlement organizations, in each province.^4^ The research team also mobilized other organizations, which maintain frequent contacts with business representatives (e.g., local Chambers of Commerce and sector organizations) to collect more responses. SMEs responded on a voluntary basis. In the end, 112 questionnaires were usable for the analysis (15 from Alberta, 21 from Saskatchewan, and 76 from Manitoba). Appendix 1 depicts respondents' experience with international immigrant labor. The SME representatives' responses are coded using the following standard Province-BX, where X is the number of the business B. AB stands for Alberta, MB for Manitoba, SK for Saskatchewan.

The participants of the integration study were selected using a different approach. First, each Prairie Province was assigned an economic function (tourism for Alberta, agriculture for Saskatchewan, and agro-industry for Manitoba). In each province, two rural communities were selected based on the fact that they host actors of the aforementioned industries, which led to the selection of Banff (B) and Cochrane (C) in Alberta, Moose Jaw (MJ) and Weyburn (W) in Saskatchewan, and Neepawa (N), and Portage la Prairie (PlP) in Manitoba. In each community, the research team aimed to interview at least three representatives of organizations welcoming international immigrants (ORG) and three international immigrants (IMM) selected based on their seniority in the community. Immigrants were permanent residents for permanent residents only can access settlement services. While representatives of welcoming organizations (Appendix 2) were selected through online searches and discussions with community leaders, international immigrants were selected through contacts with SPOs. International immigrants and organizations welcoming immigrants were selected through contacts with Local Immigration Partnerships (when present in the community) or community leaders otherwise.

## Results

This section is organized to depict the complexity of the interdependencies existing among immigrants, the materiality of their environment, and the immateriality of their social relationships with the host society. The results highlight first the challenges stemming from immigrants' adjustment to their new cultural environment. It continues with the ideas that this adjustment is facilitated by settlement organizations and complemented by a dense network of other organizations. Finally, the retention of international immigrants appears to take place when all the rural actors dialectically and positively respond to the arrival of immigrants.

### The Challenges of Landing in a Rural Place

All the participants (immigrants, immigration organizations, and employers) acknowledged that language plays a critical role in the integration process. B-IMM1 shared that he “keep[s] saying English because […] immigrants come to BV with very little English that kind of makes it hard for them to integrate and find a job or, you know, just get involved in the community.” This perspective was confirmed by 68% of interviewed employers, who identified language as a challenge for hiring immigrants. More than half of the immigrants interviewed also indicated that language can foster misunderstandings and conflicts between immigrant employees, employers, and domestic employees, which materialized, for instance, through perceived preferential treatment by the employer toward either party, the inability to take service calls on their own in different locations, prejudices from customers who refuse service from immigrants due to their language limitations, or even domestic employees' feelings of exclusion when immigrant employees speak their native language. Overall, the language barrier tended to complicate immigrants' jobs on the premise that the time needed to relay information and ensure understanding could affect productivity, a situation that was exacerbated when industry-specific knowledge was required.

A few other operating issues were mentioned by the participants as hindering one's ability to integrate. The top issue was transportation as hosting communities lacked public transportation allowing access to surrounding areas. W-IMM2 explained that “you might have a community that is only 15 min from another community, but if they don't have a way to get to that community […] we have taxis, but there isn't like a public transit […] there is not a continuous demand.” Of the immigrants interviewed, three also talked about their inability to follow through on desirable financial opportunities (e.g., mortgages, credit) because of their immigration status. Overall, adjusting to the Canadian financial system was perceived as another obstacle to integration. As B-IMM2 explained, “All these opportunities are opening up for you […] it's just so easy now with your credit cards […]. The first thing that immigrants need to learn is how to handle their finances, because aside from our daily expenses here, most of the people I know, at least from my country, we support families back home. So, if you don't handle your finances well, you're gonna go into debt.” Time management and the devaluation of immigrant's education or work credentials were also mentioned consistently.

In face of these challenges, almost every immigrant noted they had experienced major life changes as a result of their relocation. For them, changing the way they do some things was critical to being happy or successful in their current communities. Meaningful changes involved modifying the way they eat, “I can't get the food. […] And the only way you do get Jamaican food here, it's so expensive. I can't. They were selling one [bun] for $10, can you believe? (laughs). Yeah, for somebody to get Jamaican foods here, it's very expensive” MJ-IMM2; commute, “It's in Makati City, it's a business center. So we just have to ride any transportation. It's not, not the same here that we are living in the countryside. I don't have a driver's license too” N-IMM1; and exercise, “learn new things, learn about Canadian culture and its landscape and all that. So I tried different things. First, I started to take the opportunity and go bike and hike and all that […] so I can learn about the environment, about the community and meet like-minded people” (B-IMM1). Overall, the quest for happiness was perceived as their responsibility and their ability to handle change, which was qualified by adjectives such as being brave or smart, was critical: “I must, it's not need, I must make my life like I had in my country [.] I wanna feel [a] full life” (W-IMM1). They also recognized that engaging in activities such as educational advancement and upskilling were important to their state of wellness.

### Settlement Organizations at Your Service

The critical role of settlement organizations was well-recognized among immigrant interviewees. Indeed, all of them shared positive feedback about the services they had received from settlement organizations, such as B-IMM1, who explained that “if you're a newcomer in town […], when you come to Settlement Services you know that the people that work there, they have similar stories to yours, that they will understand […] that they'll try to find the best approach. So, and that's a service available in town, which is amazing.” Personnel from the organizations intended to build a privileged relationship with immigrants. Although there was no formalized procedure, they usually started with a needs assessment that aimed to individualize support and then referred immigrants to adequate experts or resource services according to identified needs. Their reliance on an extensive network of partners was embedded in their recognition of the multifaceted aspects of settlement and integration. Even though their mandate may focus on the most urgent needs encountered by immigrants after landing in rural Canada, they regularly went past their mandate, which sometimes involved using *ad-hoc* resources, “we use our volunteers to offer other people to benefit from these services” (PlP-ORG1).

The core activities of settlement organizations were to support immigrants to go through the basic administrative formalities that would facilitate their integration into the Canadian society, such as getting a Social Insurance Number and health coverage, as well as enabling immigrants to cover their basic needs, such as finding accommodation and securing a job. For a newly landed immigrant, finding a job was both a vital step to provide for the basic needs and a major challenge: “So to speak it's a survival, we were in survival mode, right? So we're here for jobs, and we don't really have time for anything else. Like, I only had one job back then but most people I know, friends I know, they hold two jobs so you don't really have time to, you know to socialize” (B-IMM2). In this regard, settlement organization personnel lavished advice to help international immigrants design CVs and write résumés that match Canadian employers' standards. They also helped immigrants identify overlooked strengths and experiences that could be valued during interviews. Interviews resonated particularly challenging for immigrants who shared making lots of efforts to soften their accent and behave according to their perception of accepted Canadian standards: “I adapt. Like it's harder at first, but then I seem to pull myself together to adapt to where I'm functioning […] It just takes time, but it happens.” (W-IMM3).

The fulfillment of these needs highlights an invisible cultural barrier that slowed down the integration process, which is well-exemplified by the international kitchens organized by W-ORG3 that enabled local Canadians to relate to newcomers' trajectories and realize the cultural differences that exist. Often tacit for most individuals of the host society, this cultural barrier was an essential background knowledge that sounded difficult to gain by immigrants. In order to overcome this barrier, settlement organization personnel attempted to build bridges between the host society and immigrants. Their goal was to facilitate the acquisition of cultural norms through experience in a safe social space. A variety of activities were organized based on available resources, people's skills, and local interests. A glimpse of these activities includes potlucks, dance nights, women nights, discussion tandems, discussion groups, and outdoor activities that all enable immigrants to value their own culture and capacities. For settlement organization personnel, building these social relationships appeared to be an essential part of immigrants' integration process. These activities tended to result in a feeling of independence stemming from a more comfortable navigation of social life in rural Canada, which sometimes could be tied to intangible statuses such as being a homeowner as PlP-IMM1 explained, “If you are able to buy a property over here (…) the country trusts you, right. If they trust you, you're part of it. (…) That's a little piece of Canada.”

However, this was not necessarily a panacea as this learning process was not neutral. The host society may not necessarily respond or may even respond negatively. In half of the communities, interviews highlighted tensions between immigrants and the host society, which, in some cases, led to the formation of ethnic enclaves or a perceived feeling of racism. For instance, employers shared that “the intellectual people we serve do not understand newcomers” (MB-B27), that “some clients are resistant to accepting service from newcomers” (MB-B64), or even that “there was a client [immigrant], his tires were punctured” (W-ORG3). This socialization could also lead to tensions within the household, which was the case for C-IMM1 whose “husband did not know English at all. [She] had to do everything, all the paperwork, and explain everything to him. They call [her] the boss. It was very difficult.” In the communities under investigation, other organizations also provided valuable support.

### A Dense Network of Supporting Organizations

During the study, a wide diversity of organizations other than settlement organizations appeared to contribute as well to the integration of international immigrants in Canada. In a more or less purposive manner, all these actors facilitated the acquisition of rural Canada background knowledge. Usually, they responded to needs that were not directly tackled by settlement organizations or added value to the services already provided by settlement organizations. Although, in some rare cases, the provision of community services was redundant to services provided to immigrants only, in most cases, the network of organizations and the referral system avoided such redundancies. Actors' motivation and intentionality varied in a great manner. For instance, PlP-ORG3, a real estate agent, facilitated access to her network of financial establishments in the perspective of sealing a deal, which, at the same time, contributed to immigrants' financial literacy. The faith-based MJ-ORG2 and immigration B-ORG3 organizations emphasized the lack of transportation in rural areas, which had resulted in informal and formal transportation services, respectively.

On the one hand, some of these complementary organizations seem to be more culture-sensitive. Often composed of former immigrants or members who acknowledge the hardship of establishing a new life in a foreign country (MJ-ORG2, MJ-ORG3, N-ORG3), these organizations provide a friendly atmosphere for sharing experience, playing down the hiccups of starting a new life in a new country, and providing mental health support and counseling to respond to immigrants' depressing loss of autonomy. For instance, it helped W-IMM3 who explained, “I didn't have my license transferred over to Canada. […] They wouldn't let me get it [the license] until I got all this stuff situated. […] So I just kinda had a little bit of depression for the first… like probably a year or so, […] just like you have that cabin fever and you're just like mopey and stuff” or N-IMM3 who explained, “actually you know what, for the first 5 months I feel depressed that it's so hard. [It was] sad for me that it's really hard to find a job. Yeah, it's really struggling that whether you like it or not, [this is now] your hometown, you need to accept this.” This supportive environment enabled sharing knowledge in culturally appropriate manners, which was not always the case in settlement organizations despite all the efforts made by the personnel to demonstrate respect to immigrants' otherness (e.g., women nights). However, these networks were also susceptible to slowing down the integration process when the refuge they provide may lock in the participants in a cultural comfort zone that was too comfortable to depart from. C-IMM1 found that “if you stay with Syrians, you won't be growing. They are still connected to each other. They could not absorb. […] It is hard for them to accept different traditions and ways of thinking.”

The workplace was another significant place for secondary socialization, which contrary to the previous organizations was much less culturally sensitive. While employers acknowledged several barriers and cultural gaps affecting the integration of international immigrants in their workforce, they also emphasized all the benefits of working with them. For instance, MB-B72 recognized that they “have managed to employ some incredibly loyal and hardworking employees who were newcomers.” Consequently, they provided services that can foster the integration of international immigrants and facilitate relationships among workers. The sometimes distant settlement organizations were not always easily accessible for immigrants who did not necessarily have a driving license or individual transportation. As a matter of fact, employers acted as substitutes that provided settlement services. For instance, employers shared doing language training, opportunities for credentialing, furthering studies, or buying tools. They also emphasized paying special attention to maintaining an inclusive work environment and regretted tensions stemming from wrongly interpreted actions among coworkers.

### Toward a Dialectical Transformation of Rural Places

Results also suggest a multilevel transformation of both the immigrants themselves, the businesses where immigrants work, and the community that host immigrants. While the interrelations happening at the workplace tended to create opportunities to engage culturally: “getting feedback in the workplace and being able to read people's [non-verbal clues] and body language help with how to communicate with different people from different cultural backgrounds” (B-IMM1), having multiple jobs, which was the case for several interviewees, prevented participation in leisure activities where the socialization process could take place in a more friendly manner. For unemployed immigrants, creating their own activities helped them engage with other newcomers and locals alike: “I was a little bored and like I needed to find something to meet people and learn about Canmore [.] I joined meetup.com. […] So, I started my own. I love walking in the mornings. So, I started Canmore Social Morning Walk” (B-IMM1). However, most interviewed immigrants did not find it easy to engage with their new cultural environment, primarily because they did not know where to make these positive connections: “I found a connection with my job, and it's fulfilling for me. That's when I started to [.] be more aware of what's going on, the services and the programs that are out there available” (B-IMM2). Interestingly, many immigrants perceived that one has to be smart and brave (C-IMM1, W-IMM1) in order to create connections or achieve a fulfilling life.

For participating employers, the valuable changes immigrants bring in the workplace are workforce stability, economic benefits, and workplace diversity, along with strong values and work ethics: “My staff is more stable. Hiring newcomers has assisted us in filling vacancies within our agency; 75% of our employees would be considered newcomers” SK-B90. For 52% of the respondents, the stability and economic benefits stemming from immigrants' capacity to fill labor shortages decreased turnover rates, reduced vacancies, and boosted employee retention while it boosted staff morale and created a sense of security to stay with the company. “[Newcomers] fill extreme labor shortages that in turn offer stability and therefore retain the Canadian workers we have on staff, particularly managers. Otherwise, they would have quit long ago” MB-B44. Economically, it allowed companies to undertake more jobs thereby increasing their sales/revenue: “I have a more stable workforce that has correlated with increased sales” MB-B26, as well as attract new customers: “We have just over 30% diverse hiring and many are new Canadians […] a main benefit is that they help my business to better reflect the changing community [.] and that keeps us relevant to the marketplace” MB-B30. This created space for immigrants to share new perspectives to create new business opportunities: “Sometimes they bring a new perspective to the store and illuminate some opportunities for us” SK-B89. Many employers (40%) mentioned immigrants' strong work ethics and problem-solving capacities: “I have hired loyal, hardworking employees, who are willing to work, and appreciate the opportunity to work” MB-B46. An attitude of other-centeredness is developed that “help[s] everyone to look beyond their own needs to consider others. That outward mindset fosters inclusiveness, gratefulness, [and] appreciation for differences of all types” MB-B19.

Results finally suggest that changes in the workplace spilled over into the community, usually through the family members that immigrants bring with them. Facilitating newcomers through employment kept rural businesses opened: “Without newcomers, we pretty much wouldn't exist” MB-B10. The settlement of immigrants “has changed my rural community in the 20-plus years since I moved here. it is a place for our children who wanted to leave to a diverse community [that has] winter festivals, tribal days, and huge national events” MB-B22. Employers' attitude toward immigrants was often translated into activities that reflect communities' attitudes of openness and welcome: “Our city helps newcomers right from the minute they inquire through to rides from the airport to accommodations, to finding jobs, to get-together evenings. The city of Morden goes over and above to welcome newcomers” MB-B10. Different community groups (e.g., churches, settlement organizations, support groups) rallied together to solve issues that affect newcomers and the community alike: “Portage Learning Centre is the best example, as they are doing an awesome job. I myself do volunteer work as a Settlement Partner with newcomers to improve their language skills and how to adapt to the new environment in life. We work on various problems as a team and try to solve them” MB-B17. One community had even established a not-for-profit to address newcomer needs: “Build a Village is an organization within the community that specifically helps refugees” MB-B20. Important to one employer AB-B111 were the “lifelong friendships” that were forged.

## Discussion

The results shed light on both immigrant integration processes and rural employers' involvement in the retention of international immigrants. The challenge of the utilization of two datasets emanating from two different research studies was overcome by the design of an original iterative data analysis process, inspired by an abductive perspective, during which the assumptions have been adjusted several times to the diversity of the materials. This process enabled us to take advantage of both the quantitative data of the employers' motivations study and the qualitative data of the rural integration study. Even though the nature of the studies was complementary, employers interviewed in the first study were not necessarily employers of immigrants in the second study. These results could be complemented with a survey addressed to immigrants landing in rural areas to better quantify the importance of dialectical relationships with immigrant organizations, employers, and other residents. Such a study would eventually lead to the identification of subgroups of immigrants based on the nature of the dialectical relationship they entertain. In addition, it would be interesting to shed more light with a qualitative analysis on the relationships that exist between rural employers and immigrant organizations to better understand sectoral opportunities and challenges.

The utilization of Berger and Luckmann's (1966) secondary socialization concept was primarily motivated by the goal to better understand the role of mediating organizations during the integration of international immigrants and their impact on immigrants' well-being. It provides an opportunity to conduct a meso-level analysis that goes beyond the multiculturalist view of acculturation, “those phenomena which result when groups of individuals having different cultures come into continuous first-hand contact, with subsequent changes in the original cultural patterns of either or both groups” (Redfield et al., 1936: p.149), and the assimilationist view of segmented assimilation, as described earlier. While the former enables a distinction between four trajectories (i.e., integration, assimilation, marginalization, and rejection), the latter enables an articulation of the influence of macro-social structures (e.g., culture, power relationships) with micro-behaviors (e.g., internalization, conformism). The meso-level stance adopted with the utilization of the concept of secondary socialization facilitates the simultaneous consideration of these aspects through interactions existing among social actors. On this specific point, the investigation of the role of food, understood as an unanimated mediating factor of acculturation, appears particularly promising. The difficulties for accessing culturally appropriated food, which appeared to be a significant factor of social inclusiveness during events such as international kitchens, resonated particularly strongly to most participating immigrants.

Immigrants' credentials, which foster successful immigration, appear devalued or unrecognized when immigrants enter Canada, compounding their ability to gain employment that matches their qualifications and experience. Employers perceive the benefits of hiring newcomers, but are limited in realizing their full potential economically. Most immigrants shared accepting the devaluation of their credentials due to personal priorities or cultural orientation. While one immigrant noted choosing between credential revaluation and mortgage payments, another looked at it as a start-over. Devaluation and non-recognition are common in both regulated and unregulated professions (Kaushik and Drolet, 2018) and are referred to as the glass ceiling, the effect of having limited socioeconomic mobility in their careers; glass floor, the stagnancy in lower wage and position jobs, and glass wall, being on the outside despite attempts to enter the building (Guo, 2013). Half of all the immigrants commented on their inability to further their education, suggesting the deep ties education may have on integration. The positive correlation between economic integration (job satisfaction) and immigrant well-being may purport that gratitude for employment does not equate to immigrants' satisfaction and fulfillment. The frustration associated with discontent may contribute to a decline in overall health and more so mental health. Seemingly, settlement organizations have intervened to match pieces of previous skills with available jobs, but there is no strategy in place to fully utilize immigrants' skills set.

Neoliberal policy reforms happening in rural parts across Canada have also started to change the social and immigration landscape. These changes have brought about challenges in the uptake and utilization of services received by immigrants, but they also pose challenges for the organizations that offer services. As highlighted by our interlocutors, nearly half of participating organizations mentioned the restrictions that are placed on them by funding bodies. Some organizations had to combine federal and provincial funding in order to cover as many people as possible, or in some rural areas, in order to offer the programs at all. Therefore, immigrants found themselves having to adapt to a system that is expected to help them integrate into their host communities (Wilson-Forsberg, 2015). Immigration appears not to be a humanitarian endeavor, but rather an economic one, which consists of selecting applicants with training, work experience, language ability, and investment potential to make a positive contribution to the provincial economy (Carter et al., 2008). While the value an immigrant brings is hardly understood, even through a nominee program, the emphasis of current immigration policies seems to focus on local actors' economic needs. An aspect of this deprivation can be immobility, which leaves many people stranded within local, rural communities and forces others to employ complex strategies to access facilities and services that are not readily accessible (Milbourne and Kitchen, 2014).

## Conclusion

In this article, we explored the complexity of international immigrants' well-being during their integration in Canada with a focus on economic integration. We blended the results of two independent researches, respectively investigating employers' motivations to hire immigrants from a quantitative point of view and immigrants' integration from a qualitative point of view in the Prairie Provinces in Canada. While the quantitative data about employers' motivations support the idea that immigrants' well-being depends on intertwined social processes taking place in rural communities, the qualitative data provide a deeper understanding of both the challenges faced by immigrants and the support provided by various stakeholders of immigrants' integration.

These results emphasize the efforts that are made by rural stakeholders to facilitate immigrants' integration to the extent of their capacities. While the results confirm that economic integration is critical for immigrants' well-being to provide for themselves and their families, they also highlight the importance of economic integration for social and cultural well-being. In rural communities where resources can be scarce, immigrant's well-being is supported by the relationships immigrants develop within their community and magnified by the interdependencies that can emerge. In other words, the results suggest that a successful immigration, which would see international immigrants, settle, integrate, and remain in rural areas because they feel well, relies on a dialectical transformation of rural areas where secondary socialization of both immigrants and the hosts would take place.

The highlight of mediating organizations' critical roles for international immigrants' well-being leads to several questions. A closer look at interactions among mediating organizations could enhance the understanding of both international immigrants and organizations' needs. The governance of such complex, diversified, and place-based networks is a critical stake. The Local Immigration Partnerships initiative, which is based on the acknowledgment of networked organizations, is likely to benefit immigrants. While there is a risk that a too tight network of organizations may lack flexibility, thereby inducing immigrants' internalization of the dominant culture without having the possibility to share meaningfully their own identities, improving networking appears to facilitate the mobilization of local capacities. Understanding the dynamics of such networks could also help to design industrial and agricultural rural policies based on the idea of pooled resources shared within a network, which could enhance the development of remote rural areas where low density and long distances structure any initiative.

## Data Availability Statement

Of the original contributions collected during the study, significant ones are included in the article. Further inquiries can be directed to the corresponding author.

## Ethics Statement

The studies involving human participants were reviewed and approved by Brandon University Research Ethics Committee. The patients/participants provided informed consent to participate in this study.

## Author Contributions

JD participated in the data collection, data analysis, and article writing. SS led the research, participated in the data collection, data analysis, and article writing. MA led the research, designed the research, participated in the data collection, data analysis, and article writing. All authors contributed to the article and approved the submitted version.

## Conflict of Interest

The authors declare that the research was conducted in the absence of any commercial or financial relationships that could be construed as a potential conflict of interest.

## Footnotes

^1^Although the official label would be temporary residents (travellers, international students, and temporary workers), the scope of this article does not concern travellers and international students. The exclusion of these two subclasses of individuals facilitates the framing of the discussion of the results.

^2^The Citizenship branch was “responsible for supporting governmental and nongovernmental agencies interested in facilitating the adjustment and integration of newcomers and in developing a greater consciousness of the privileges and responsibilities of citizenship” (Canada Council for Refugees, 2000: p.19).

^3^The definition of visible minorities follows the federal Employment Equity Act of Canada. For more information, please visit https://laws-lois.justice.gc.ca/eng/acts/E-5.401/

^4^The research team worked hand in hand with the provincial umbrella organizations (AAISA in Alberta, SAISIA in Saskatchewan, and MANSO in Manitoba), which shared the survey link to their respective SPOs.

## Appendix

**Appendix 1:** Characteristics of the SME sample.

**Descriptor**	**Survey responses from SME owners**				
	**Responses**	**Average**	**Median**	**Min.**	**Max.**
Number of employees	62	68	20	0	492
Number of newcomers employed	57	23	7	0	200
1st year a newcomer was employed	50	2010	2011	1995	2,017

**Appendix 2:** Characteristics of participating organizations.

**Province**	**Community**	**Code**	**Organization**	**Role**
Alberta	Banff	B-ORG1	Bow Valley Immigration Partnership	Immigration
Alberta	Banff	B-ORG2	Bow Valley Parent Link	Parenting
Alberta	Banff	B-ORG3	Bow Valley Settlement Services	Immigration
Alberta	Cochrane	C-ORG1	Alberta Works	Employment
Alberta	Cochrane	C-ORG2	Rocky View Immigrant Services	Immigration
Alberta	Cochrane	C-ORG3	Rocky View School Division	Education
Saskatchewan	Moose Jaw	MJ-ORG1	Hillcrest Church	Culture
Saskatchewan	Moose Jaw	MJ-ORG2	Islamic Association of Saskatchewan	Culture
Saskatchewan	Moose Jaw	MJ-ORG3	Moose Jaw Multicultural Council	Culture
Saskatchewan	Weyburn	W-ORG1	Settlement Workers in Schools	Education
Saskatchewan	Weyburn	W-ORG2	Southeast College	Education
Saskatchewan	Weyburn	W-ORG3	Southeast Newcomer Services	Immigration
Manitoba	Neepawa	N-ORG1	Town of Neepawa	Community management
Manitoba	Neepawa	N-ORG2	Neepawa and Area Immigrant Settlement Services	Immigration
Manitoba	Neepawa	N-ORG3	St. Dominic's Church	Culture
Manitoba	Portage la Prairie	PlP-ORG1	Portage Learning and Literacy Centre	Immigration
Manitoba	Portage la Prairie	PlP-ORG2	Central Mental Health Association	Health
Manitoba	Portage la Prairie	PlP-ORG3	Royal le Page Realty	Housing
